# Analysis of *Dipylidium caninum* tapeworms from dogs and cats, or their respective fleas: Part 2. Distinct canine and feline host association with two different *Dipylidium caninum* genotypes

**DOI:** 10.1051/parasite/2018029

**Published:** 2018-05-28

**Authors:** Frédéric Beugnet, Michel Labuschagne, Christa de Vos, Dionne Crafford, Josephus Fourie

**Affiliations:** 1 Boehringer Ingelheim Animal Health, 29 Av T. Garnier, 69007 Lyon France; 2 Clinomics, P.O. Box 11186, Universitas, Bloemfontein, 9321 South Africa; 3 Clinvet, P.O. Box 11186, Universitas, Bloemfontein, 9321 South Africa

**Keywords:** *Dipylidium caninum*, *Ctenocephalides felis*, dogs, cats, genotypes, host association

## Abstract

Initial investigations suggested the existence of two distinct genotypes of *Dipylidium caninum* from infected cat fleas (*Ctenocephalides felis*). One genotype was found almost always (> 95%) in fleas collected from, and proglottids shed by, domestic dogs. The other was found almost always (> 95%) in fleas collected from, and proglottids shed by, domestic cats. Molecular investigations (Part 1, in this journal) confirmed the presence of two distinct genotypes. Due to the apparent host association observed, these were referred to as the “*D. caninum* canine genotype” and the “*D. caninum* feline genotype”. The current article reports on an *in vivo* experimental infection study assessing the host-parasite interaction for each genotype. Mixed infections with the two genotypes in both dogs and cats were conducted. The specific genotyping of proglottids allowed us to assess the specific prepatent periods, prolificity, and longevity of each genotype in dogs *versus* cats. The possible hybridisation was also studied through molecular evaluation of the proglottids expelled by infected dogs and cats. Results demonstrate a clear distinct host interaction. The canine *D. caninum* genotype occurred at a higher frequency in dogs, with a shorter prepatent period and a longer lifespan; and the feline genotype occurred at a higher frequency in cats, with a shorter prepatent period and a longer lifespan. The absence of any hybrids in the mixed infections of both dogs and cats confirm the hypothesis of two distinct genotypes, suggesting the possibility of two distinct species within *Dipylidium caninum*.

## Abstract (fr)

Des investigations initiales ont suggéré l’existence de deux génotypes distincts au sein de *Dipylidium caninum* issus de puces infectées (*Ctenocephalides felis*). Un génotype est trouvé dans plus de 95 % des cas chez des puces ou des proglottis collectés sur des chiens. L’autre est trouvé dans plus de 95 % des cas sur des puces collectées sur des chats ou des proglottis éliminés par les chats. Les investigations moléculaires publiées (Partie 1, dans ce journal) ont confirmé l’existence de ces deux génotypes. Du fait de l’apparent tropisme d’hôte, ces deux génotypes sont désignés comme génotype canin et génotype félin. Le présent article présente les résultats d’infestations expérimentales ayant pour objectif d’étudier l’interaction hôte-parasite pour chaque génotype. Des infestations mixtes ont été réalisées avec les deux génotypes chez des chiens et des chats. Le génotypage spécifique a permis d’étudier les périodes prépatentes, la prolificité et la longévité de chaque génotype chez chaque hôte. La possible hybridation a aussi été étudiée par évaluation moléculaire des proglottis éliminés par les chiens et les chats infestés. Les résultats ont démontré une interaction hôte-parasite bien distincte. Le génotype canin de *D. caninum* a une fréquence plus élevée chez les chiens, avec une période de prépatence plus courte et une durée de vie plus longue, et le génotype félin a une fréquence plus élevée chez les chats, avec une période prépatente plus courte et une durée de vie plus longue. L’absence de tout hybride dans les infections mixtes des chiens et des chats confirme l’hypothèse de deux génotypes distincts, suggérant la possibilité de deux espèces distinctes au sein de *Dipylidium caninum*.

## Introduction

*Dipylidium caninum sensu lato* is an important cestode parasite with a worldwide distribution, as is evident from surveys performed in wild canids and felids, domestic cats, domestic dogs, or concurrent surveys assessing both domestic cats and dogs [1,3,4,6,8–11,13–19,21,25,26,28,30,32,34–37,40,41,43–48]. Apart from infecting both canids and felids, this cestode may also occasionally infect humans [2,24,42].

The intermediate hosts for this parasite are the cat and dog fleas (*Ctenocephalides felis* and *Ctenocephalides canis*, respectively), as well as the dog and cat chewing lice, *Trichodectes canis*, and *Felicola subrostratus*, respectively [31]. Due to its worldwide distribution, and its ability to infest dogs and cats, the cat flea, *C. felis*, is considered to be the main intermediate host [5,12,27,46]. Flea larvae ingest *D. caninum* eggs, with the rate of development in the flea greatly affected by temperature [38,39]. When adults fleas infected with suitably developed metacestodes are ingested by the canine or feline host, the parasite establishes in the small intestine. Here it develops to an adult tapeworm, with shed proglottids visible in faeces from between 17 and 19 days after infection [22,23].

Beugnet *et al.* [8] investigated the occurrence of *D. caninum* in fleas from client-owned cats and dogs in Europe, using a new PCR detection assay. The results indicated that easy and regular *Dipylidium* sp. re-infections of both cats and dogs in European households were likely. Thus, for the first time, the spread of *D. caninum* between fleas on dogs and cats was confirmed throughout Europe. In this European survey, 2.23% of 1969 cat fleas collected from cats were found to be infected by *Dipylidium* sp. larvae, compared to 5.2% of 732 cat fleas collected from dogs and 3.1% of 2828 dog fleas collected from dogs. Preliminary analyses performed during this survey, indicated genetic differences between *D. caninum* metacestodes in fleas collected from dogs and cats, respectively. Low, in 2017, suggested the presence of two clades within *D. caninum* species [31]. Labuschagne *et al.*, 2018, using the DNA extracted from the initial flea collect from dogs and cats in Europe [8], and adding new fleas collected in the United States, as well as *Dipylidium* proglottids from Europe, Africa, and Asia, demonstrated the existence of the two distinct genotypes [29]. The initial genetic analysis started in 2012 during an epidemiological survey assessing the infection rate of fleas by *Dipylidium caninum* using a new PCR probe [8]. In the recent paper, Labuschagne *et al.*, 2018, established a correspondence between the host origin of *Dipylidium-*infected fleas and the genotype. They demonstrated that the genotypes are not related to geography but to hosts. The so-called feline genotype of *D. caninum* was found almost exclusively in *C. felis* collected from cats (95.1%), whereas the so-called canine-genotype was found almost exclusively in *C.felis* collected from dogs (97.3%), and was the only one observed in *C. canis* fleas (100%) [29]. The authors also confirmed that the *Dipylidium* DNA collected by Low *et al.*, (2017) [31], from cat fleas and cat lice collected from cats belong to the feline genotype, and that the *Dipylidium caninum* collected by East *et al.*, (2013) [17], also belong to the same feline genotype [29,31]. The *Dipylidium* from the two genotypes are kept in dogs and cats at Clinvet, Bloemfontein, South Africa, allowing the present study.

This paper reports on an *in vivo* experimental infection study, designed to investigate potential host association with reference to the canine and feline *D. caninum* genotypes [29].

The objectives of this study were thus two-fold: firstly, to establish whether the two *D. caninum* genotypes show distinct host interaction, *i.e.* prepatent period, longevity, and rate of infection; and secondly, to establish whether the genotypes could have sexual reproduction during mixed infection in either dogs or cats.

## Materials and Methods

### Ethics

The study was approved by the Institutional Animal Care and Use Committee (IACUC). The study conformed to the principles defined and explained in the European Convention for the Protection of Vertebrate Animals used for Experimental and Other Scientific Purposes and its appendix. In addition, the authors have involved the minimum number of animals in the experimental infection study for the purpose of adequate experimental infection model validation. Animals were observed daily for general health, with physical examinations performed by a veterinarian to ensure suitability for inclusion in the study. Throughout the study, the health of the animals was monitored by veterinary personnel. No abnormal clinical signs were observed during either clinical examinations or daily health observations. As a result, none of the animals required concomitant therapy or veterinary care during the study. After termination of the animal phase, the animals received the necessary concomitant therapy (deworming based on praziquantel oral administration), after which they were returned to the Clinvet colony holding facility in order to undergo a resting period.

### Study design

**Figure 1:**
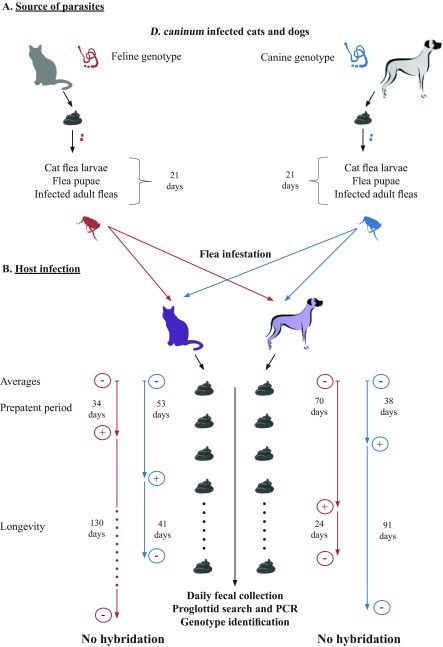
Graphical presentation of the experimental study on Dipylidium caninum genotypes and host association.

The study was designed as a parallel group, non-blinded, randomised, single-centre study, to determine the efficiency in infecting dogs and cats with two different *D. caninum* genotypes (Figure 1). The *D. caninum* were sourced from donor cats or dogs, and served to infect fleas. The study was based on an experimental flea infestation model previously published [7,22], in combination with the newly developed PCR hydrolysis probe assay [29].

### Animal details

Three dogs (group 1) and three cats (group 2), all females, were included in the study. The dogs were all beagles, 10 months of age and weighed between 10.20 kg and 11.60 kg. The cats were all domestic shorthair, with ages 7, 9 and 27 months respectively, and body weight ranging between 1.90 kg and 2.92 kg.

### Experimental model overview

Cats and dogs were infected concomitantly with both the canine and feline *D. caninum* genotypes, by means of skin infestation with *D. caninum-*infected *C. felis* fleas.

See Fourie *et al.* [22,23] as well as Beugnet *et al.* [7] for a detailed description of the experimental infection model employed.

The primary criteria for model validation was a positive result on PCR hydrolysis probe genotyping performed on faeces or positive identification of a *D. caninum* proglottid collected during macroscopic examination of cages and/or faeces [29]. While both methods constituted confirmation of *D. caninum* infection, PCR hydrolysis probe genotyping also constituted confirmation of the genotype and hence allowed evaluation of potential genotype host associations. PCR hydrolysis probe genotyping also allowed observation of hybrid DNA patterns. A secondary criterion considered was the duration of proglottid shedding.

### Flea infestations and related actions

A first step was to breed two batches of fleas, from eggs to pupae, during approximately 21 days on a flea-rearing medium containing *Dipylidium* proglottids originating from infected donor cats and dogs with their respective *Dipylidium* genotype.

A second step before flea infestation of dogs and cats was to perform PCR hydrolysis probe genotyping analyses on the newly emerged fleas to confirm their infection by *D. caninum* larvae (as well as the *D. caninum* genotype).

In addition, a sample of 30 fleas from each batch used was dissected and examined microscopically to determine the prevalence of infection with *D. caninum* metacestodes, as well as their level of development. With reference to the latter, some organisation of the hooklets had to be evident in at least one of the metacestodes present.

Fleas were killed by freezing them, after which they were dissected with the aid of a dissection microscope using two needles. One needle was used to pin the flea down by the thorax, and the other to cut open the tip of the abdomen. Contents were squeezed out using the needle. Metacestodes, if present, were counted and stage of development noted.

The third step consisted in the infestation of dogs and cats with live fleas from the two batches. As fleas containing respective feline and canine genotypes were placed on the animals, it was necessary to have batches with similar *Dipylidium* sp. infection rates for each infestation.

Thus, after establishing metacestode infection rates, the batches were “diluted” by addition of fleas from a laboratory *C. felis* flea strain known not to be infected with *D. caninum*, in order to achieve infection rates that were similar between the two *Dipylidium* infected flea batches.

Flea infestations were performed on Days 0, 13 and 28. Each cat/dog was skin infested with 200 fleas, including 100 infected fleas (50 with each *D. caninum* genotype). Animals were allowed to groom freely. Dogs and cats were housed individually during the 168 days of the animal phase.

At Day 56, in order to kill fleas, each animal was treated with an ectoparasiticide (Frontline Plus^®^ for cats and NexGard^®^ for dogs), according to label instructions.

### Proglottid collection and analyses

Macroscopic examination of faeces and the individual cages for shed proglottidswas performed at least twice weekly from Days 21 (estimated end of pre-patent period following first flea infestation on Day 0) to Day 168. Collected proglottids were subjected to DNA isolation using a commercial kit. Isolated DNA was subjected to specific PCR amplification of the 28S rDNA region as described by Beugnet *et al.* [8], followed by genotyping using hydrolysis probes specific for each genotype [29].

PCR hydrolysis probe-based genotyping was used to discriminate between the two identified genotypes exhibiting specific associations towards dogs and cats.

All proglottids were also screened for hybridization using a hybridization probe-based DNA genotyping qPCR assay [29].

### Statistical analysis

Seven day periods were used to define “weeks”, as was employed in statistical analyses (Table 1). Weeks were defined by the Investigator based on the pre-patent period of *D. caninum*, and hence the anticipated commencement of proglottid shedding.

**Table 1:** Definition of “weeks” (as used in statistical analyses) according to study day periods.

Study day period	Week
19 to 23	1
26 to 30	2
33 to 37	3
40 to 44	4
47 to 51	5
54 to 58	6
61 to 65	7
68 to 72	8
75 to 79	9
82 to 86	10
89 to 93	11
96 to 100	12
103 to 107	13
110 to 114	14
117 to 121	15
124 to 128	16
131 to 135	17
138 to 142	18
145 to 149	19
152 to 156	20
159 to 163	21
166 to 168	22

The validity of the experimental model was confirmed based on the positive identification of *D. caninum* proglottids in faeces.

The success of infection by the *D. caninum* genotypes was measured by the number of dogs/cats being infected by each genotype, respectively.

For real-time PCR results, canine and feline genotypes were presented descriptively for each three-week interval and overall period. Differences between these genotypes for each interval were compared using a Chi-square test. The level of significance of the formal tests was set at 5% and all tests were two-sided.

The pre-patent period was defined as the number of days from first flea infestation (Day 0) to the first PCR-positive test in proglottids collected from faeces.

The duration of infestation for each *D. caninum* genotype (worm longevity) was defined as the total number of days where the infestation was regarded as successful, as confirmed by the presence of *D. caninum* proglottids in faeces and their identification by PCR.

The rate of success, the pre-patent period and the duration of infestation were presented descriptively for cats and dogs, for both the feline and canine *D. caninum* genotypes respectively, at each assessment time point.

The rate of success was presented using frequencies and percentages, while the duration of the pre-patent period and the duration of infestation were presented using summary statistics (mean, standard deviation, median, minimum and maximum).

SAS Version 9.3 TS Level 1M2 was used for all the statistical analyses.

With reference to sample size, three dogs and three cats were used in this study, which was considered adequate for experimental model method validation using different genotypes. The statistical unit was the individual animal.

### Efficiency of the *in vivo* experimental model

The model was regarded as effective if host animals challenged with fleas infected with both feline and canine genotypes of *D. caninum* became infected, as confirmed by expulsion of proglottids and verified by PCR, with either or both genotypes.

## Results

### Metacestode infection rates

The metacestode infection rates in flea batches placed on animals (obtained through batch dilution with uninfected fleas as described previously), are tabulated in Table 2. Actual metacestode infection rates in the flea batches employed for host infestations ranged between 10% and 33.3%.

**Table 2:** Summary of metacestode infection rates of the fleas used, prior to each infestation.

Infestation Day	Metacestode infection in the original flea batches (%)		Age of the flea batches	Comments
			
	Feline genotype	Canine genotype		
0	40	33.3	13 days	Feline genotype diluted to 33.3% infection rate by adding uninfected fleas
13	13.3	56.7	20 days	Canine genotype diluted to 13.3% infection rate
28	10	60	20 days for the canine genotype 21 days for the feline genotype	Canine genotype diluted to 10% infection rate

### Infection success rates

The rates of *D. caninum* infection success are presented descriptively for cats and dogs, for both the feline and canine *D. caninum* genotypes respectively, at each assessment time point, in Table 3.

**Table 3:** Rate of *Dipylidium caninum* infection success (positive animals based on presence of proglottids and positive RLFP results) expressed as frequencies and percentages for the time periods assessed.

Type	Weekly interval	Group 1 (dogs)	Group 2 (cats)
			
		Rate	Rate
Canine genotype	Week 2 to 4	2 / 3 (66%)	1 / 3 (33%)
	Week 5 to 7	3 / 3 (100%)	2 / 3 (66%)
	Week 8 to 10	2 / 3 (66%)	3 / 3 (100%)
	Week 11 to 13	3 / 3 (100%)	2 / 3 (66%)
	Week 14 to 16	3 / 3 (100%)	2 / 3 (66%)
	Week 17 to 19	1 / 3 (33%)	3 / 3 (100%)
	Week 20 to 22	2 / 3 (66%)	3 / 3 (100%)
	Total (Week 2 to 22)	3 / 3 (100%)	3 / 3 (100%)
Feline genotype	Week 2 to 4	2 / 3 (66%)	3 / 3 (100%)
	Week 5 to 7	1 / 3 (33%)	3 / 3 (100%)
	Week 8 to 10	1 / 3 (33%)	3 / 3 (100%)
	Week 11 to 13	–	3 / 3 (100%)
	Week 14 to 16	–	3 / 3 (100%)
	Week 17 to 19	2 / 3 (66%)	3 / 3 (100%)
	Week 20 to 22	1 / 3 (33%)	3 / 3 (100%)
	Total (Week 2 to 22)	3 / 3 (100%)	3 / 3 (100%)

Group 1: Dogs were infected with the canine and feline *D. caninum* genotypes by means of topical infestation of infected *C. felis* fleas. Group 2: Cats were infected with the canine and feline *D. caninum* genotypes by means of topical infestation of infected *C. felis* fleas.

### *Dipylidium* sp. infection in dogs

Infections with the canine *D. caninum* genotype were first observed in all three dogs from Week 5 to 7, with observed infections persisting throughout the study period, while infection with the feline genotype was not observed in all three dogs during that period. Infection with the feline genotype in dogs was observed in 2 out of 3 dogs positive from Week 2 to 4, and then again from Week 17 to 19. However, considering the total period, the three dogs did become infected with the feline strain.

### *Dipylidium* infection in cats

Infections with the feline genotype were first observed in all three cats from Week 2 to 4, with observed infections persisting throughout the study period, while infection with the canine genotype was not observed in all cats during that period. Infections with the canine genotype were first observed in all three cats in group 2 from Week 8 to 10, and then again from Week 17 to 22.

### Genotyping results

Hydrolysis probe-based genotyping results are presented in Table 4a (dog group) and Table 4b (cat group). These results confirmed that the canine genotype had a higher frequency of occurrence in dogs, while the feline genotype had a higher frequency of occurrence in cats.

**Table 4a:** Hydrolysis probe-based genotyping result frequency counts (*Dipylidium caninum* feline and canine genotypes) in dogs (group 1).

Time point	*Dipylidium caninum* genotype		Failed reaction n / N (%)
		
	Canine n / N (%)	Feline n / N (%)	
Week 2 to 4	121 / 146 ( 82.9)	8 / 146 (5.5)	17 / 146 (11.6)
Week 5 to 7	214 / 332 ( 64.5)	102 / 332 (30.7)	16 / 332 (4.8)
Week 8 to 10	48 / 49 ( 98.0)	1 / 49 (2.0)	
Week 11 to 13	23 / 27 ( 85.2)	–	4 / 27 (14.8)
Week 14 to 16	11 / 14 ( 78.6)	–	3 / 14 (21.4)
Week 17 to 19	2 / 31 (6.5)	3 / 31 (9.7)	26 / 31 (83.9)
Week 20 to 22	13 / 17 (76.5)	1 / 17 (5.9)	3 / 17 (17.6)
Total (Week 2 to 22)	432 / 616 (70.1)	115 / 616 (18.7)	69 / 616 (11.2)

Group 1: Dogs were infected with the canine and feline *D. caninum* genotypes by means of topical infestation with infected *C. felis* fleas.

**Table 4b:** Hydrolysis probe-based genotyping result frequency counts (*Dipylidium caninum* feline and canine genotypes) for cats (group 2).

Time point	*Dipylidium caninum* genotype		Failed reaction n / N (%)
		
	Canine n / N (%)	Feline n / N (%)	
Week 2 to 4	1 / 71 (1.4)	65 / 71 (91.5)	5 / 71 (7.0)
Week 5 to 7	7 / 168 (4.2)	152 / 168 (90.5)	9 / 168 (5.4)
Week 8 to 10	14 / 157 (8.9)	131 / 157 (83.4)	12 / 157 (7.6)
Week 11 to 13	7 / 199 (3.5)	154 / 199 (77.4)	38 / 199 (19.1)
Week 14 to 16	4 / 228 (1.8)	171 / 228 (75.0)	53 / 228 (23.2)
Week 17 to 19	35 / 526 (6.7)	450 / 526 (85.6)	41 / 526 (7.8)
Week 20 to 22	27 / 387 (7.0)	349 / 387 (90.2)	11 / 387 (2.8)
Total (Week 2 to 22)	95 / 1736 (5.5)	1472 / 1736 (84.8)	169 / 1736 (9.7)

Group 2: Cats were infected with the canine and feline *D. caninum* genotypes by means of topical infestation with infected *C. felis* fleas.

Results (*p*-values) after statistically comparing the genotyping results for the two groups (Chi-square analysis), with reference to *D. caninum* genotype employed (either canine or feline), are presented in Table 5.

**Table 5:** Statistical comparison of the cat and dog groups in terms of *D. caninum* genotypes.

Comparison	Type	Time point	*p*-value
Group 1 with group 2	Canine genotype	Week 2 to 4	<0.0001
		Week 5 to 7	<0.0001
		Week 8 to 10	<0.0001
		Week 11 to 13	<0.0001
		Week 14 to 16	<0.0001
		Week 17 to 19	0.9649
		Week 20 to 22	<0.0001
		Total (Week 2 to 22)	<0.0001
	Feline genotype	Week 2 to 4	<0.0001
		Week 5 to 7	<0.0001
		Week 8 to 10	<0.0001
		Week 11 to 13	<0.0001
		Week 14 to 16	<0.0001
		Week 17 to 19	<0.0001
		Week 20 to 22	<0.0001
		Total (Week 2 to 22)	<0.0001

*p*-value: Chi-square test Group 1: Dogs were infected with the canine and feline *D. caninum* genotypes by means of topical infestation with infected *C. felis* fleas. Group 2: Cats were infected with the canine and feline *D. caninum* strains by means of topical infestation with infected *C. felis* fleas.

With the exception of Week 17 to 19, dogs and cats differed significantly with regard to the feline and canine *D. caninum* genotype frequency of occurrence.

### Durations of pre-patent period

Durations of the pre-patent period are presented in Table 6a (dog group) and Table 6b (cat group), using summary statistics (mean, standard deviation, median, minimum and maximum). In dogs, the average pre-patent period was shorter for the canine genotype (*i.e.* 38 days) compared to the feline genotype (70 days), while the opposite was true in cats (34 days for feline genotype *versus* 53 days for canine genotype). With 3 animals in each group, these differences were not significant.

**Table 6a:** Individual and summary statistics of pre-patent periods (in days) for dogs (group 1).

Animal ID Statistics	Canine strain	Feline strain
5A8 8F3	26	29
5A9 67F	27	30
697 FFA	61	152

n	3	3
Mean	38.0	70.3
SD	19.92	70.73
CV %	52.4	100.6
GeoMean	35.1	51.2
Median	27.0	30.0
Minimum	26	29
Maximum	61	152

* *p* = 0.4884 (no significant difference between canine and feline genotypes) The prepatent period is defined as the number of days from first flea infestation to the first PCR+ test in faeces. Group 1: Dogs were infected with the canine and feline *D. caninum* genotypes by means of topical infestation of infected *C. felis* fleas.

**Table 6b:** Individual and summary statistics of pre-patent periods (in days) for cats (group 2).

Animal ID Statistics	Canine strain	Feline strain
5A2 F40	35	40
5CA 06E	70	27
869 F1E	54	35

n	3	3
Mean	53.0	34.0
SD	17.52	6.56
CV %	33.1	19.3
GeoMean	51.0	33.6
Median	54.0	35.0
Minimum	35	27
Maximum	70	40

*p*** **=** **0.1534 (no significant difference between canine and feline genotypes) The prepatent period is defined as the number of days from first flea infestation to the first PCR+ test in faeces. Group 2: Cats were infected with the canine and feline *D. caninum* genotypes by means of topical infestation of infected *C. felis* fleas.

### Durations of infestation

Durations of infestation are presented descriptively for cats and dogs, for both the feline and canine *D. caninum* genotypes, respectively in Table 7a (group 1) and Table 7b (group 2). In dogs, the observed infection with the canine genotype persisted longer compared to the feline genotype (91 days *versus* 24 days), while the opposite was true for cats (130 days for the feline genotype compared to 41 days for the canine one). These differences were significant.

**Table 7a:** Individual and summary statistics of duration of *Dipylidium* infection (in days) for dogs (group 1).

Animal ID Statistics	Canine strain	Feline strain
5A8 8F3	102	38
5A9 67F	115	33
697 FFA	58	1

n	3	3
Mean	91.7	24.0
SD	29.87	20.07
CV %	32.6	83.6
GeoMean	88.0	12.8
Median	102.0	33.0
Minimum	58	1
Maximum	115	38

*p* = 0.0312 (significant difference between canine and feline genotypes) The duration of infestation is defined as the total number of days where the infestation was regarded as successful as confirmed by RLFP results. Group 1: Dogs were infected with the canine and feline *D. caninum* genotypes by means of infestation with infected *C. felis* fleas.

**Table 7b:** Individual and summary statistics of duration of *Dipylidium* sp. infection (in days) for cats (group 2).

Animal ID Statistics	Canine strain	Feline strain
5A2 F40	55	123
5CA 06E	26	136
869 F1E	43	132

n	3	3
Mean	41.3	130.3
SD	14.57	6.66
CV %	35.3	5.1
GeoMean	39.5	130.2
Median	43.0	132.0
Minimum	26	123
Maximum	55	136

*p* = 0.0007 (significant difference between canine and feline genotypes) The duration of infestation is defined as the total number of days where the infestation was regarded as successful as confirmed by RLFP results. Group 2: Cats were infected with the canine and feline *D. caninum* genotypes by means of infestation with infected *C. felis* fleas.

### Hybridization

No sign of hybridization between *D. caninum* genotypes was detected for any of the proglottid specimen samples analyzed. This demonstrates that no hybrid proglottid-containing eggs were observed, despite the six mixed infections (three in dogs, three in cats) allowing potential sexual reproduction between adult *Dipylidium* sp. in the intestine.

## Discussion

The experimental infection model based on infected flea challenges has previously been used with great success in several efficacy studies [7,22,23]. The molecular characterization of *D. caninum* isolates collected from dogs, cats, and in infected fleas collected either from dogs or cats enabled the identification of two distinct genotypes that clearly differ from each other [29]. Previous studies had also suggested the existence of different genetic profiles, or suggested that there could be clades or even different species under the name *Dipylidium caninum* [31].

East *et al.*, 2013, collected *Dipylidium caninum* proglottids from six spotted hyena [17]. They used one of these samples to obtain 12S rRNA fragments (314 bp and 1176 bp). When comparing their 314 bp sequence data with two published *D. caninum* sequences of the same fragment, they obtained a high (99%) similarity to one sequence from Europe (accession number L49460.1) but a considerably lower similarity (89%) to one sequence from Asia (accession number AB031362.1). When they compared the available 1176 bp sequence (accession number KF202097) to their similar fragment from *D. caninum,* they obtained a relatively low similarity (89%). By looking at their sequences and comparison to the complete mitochondrial (mt) sequences of the *D. caninum* feline genotype (MG587892), Labuschagne *et al.*, obtained 99.1% identity between the *D. caninum* isolated from the hyena (KF202097) and the *D. caninum* feline genotype (MG567892) isolated from a cat [29]. When comparing to the mt genome of the *Dipylidium* dog genotype, there was only around 88.5% identity [29]. More recently, Low *et al.*, (2017) [31], collected ectoparasites from dogs and cats in Malaysia. In this study, *Ctenocephalides felis* (92 specimens) and *Felicola subrostratus* (30 specimens) were collected from 20 cats. PCR amplification utilizing the primers published in 2014 [8] was performed for the 28S rRNA gene region of *Dipylidium*. Low *et al.* also characterized the positive samples with a 12S rRNA gene amplification [31]. They found that the representative 28S rRNA sequence isolated from their flea and louse specimens (GenBank accession no. KY751956) demonstrated 95% sequence similarity with that of *D. caninum* (GenBank accession no. AF023120), and they suggested the existence of two distinct clades within *Dipylidium caninum*. They concluded that their 12S rRNA sequences (GenBank accession no. KY751955) were identical to the spotted hyena isolate from East *et al.* (GenBank accession no. KF202097) [31]. Labuschagne *et al.* compared the 12S mt rDNA sequence of the feline and canine genotypes to the *D. caninum* 12S mt rDNA sequences used by Low *et al.* [29]. The *Dipylidium* DNA isolates collected from cat fleas and cat louse from cats in Malaysia were identical to the *D. caninum* feline genotype [29]. The hypothesis drawn by Low *et al.* [31] on the existence of two clades is thus confirmed by the work of Labuschagne *et al.*, the proposed clades corresponding to the canine and feline *Dipylidium* genotypes [29].

These two genotypes are not related to geographical origin, as they were found on all continents (*i.e.* North America, Europe, Asia, and Africa), but clearly to their host origin, dogs or cats (and hyena). Nevertheless, the specificity is not absolute, as we were able to infect cats and dogs with both genotypes during the present experimental study. Labuschagne *et al.*, studying the fleas collected in 2012 [8], indicated that around 10% of the cat fleas collected on cats and 2% of the cat fleas collected on dogs, were infected with the other genotype than the host-genotype. The common presence of both cats and dogs in the same households, being infested by the same flea species (*i.e.* *Ctenocephalides felis*), could explain the infection of cats and dogs by both genotypes, but the different observed prevalences suggested biological adaptation, hence the decision to conduct the present study. On the other hand, *C. canis* and *P. irritans* fleas being more specific to dogs, 100% of the infected fleas were found to harbour the canine genotype of *Dipylidium caninum* [29].

The results obtained during the experimental infections demonstrated significant biological variations between the two genotypes in regard to their host association. The pre-patent periods were significantly shorter for the canine genotype in dogs and the feline genotype in cats, respectively. The duration of proglottid shedding (*i.e.* patent period or longevity) was significantly longer for the canine genotype in dogs and the feline genotype in cats, thus confirming biological variations and the host specificity for each genotype. The canine *D. caninum* genotype occurred at a significantly higher frequency in dogs, and the feline genotype at a significantly higher frequency in cats. Nevertheless, the host tropism was not absolute as both canine and feline genotypes were diagnosed in cats and dogs, respectively.

Even though Cyclophyllidea cestodes are hermaphrodites and present auto-fertilization, cross-fertilization is described in the presence of several adults at the same place [20,33]. Under our experimental conditions, despite mixed infections, no hybrid DNA was observed in single proglottids, demonstrating the absence of hybrid eggs.

Genomic and mitochondrial sequencing, combined with an *in vivo* experimental study and novel PCR hydrolysis probe genotyping assay, demonstrated that the two distinct *D. caninum* genotypes [29] present significant biological differences with a specific host association. A species is classically defined by individuals being able to reproduce together. The absence of hybrid eggs raises the question of the species level of each *Dipylidium caninum* genotype. *Dipylidium caninum* Linnaeus 1758 has originally been described in dogs. Another study is planned to assess the possible presence of morphological differences in addition to the genetic and biological observations. The current results, on both the genetic and the biological aspects, raise the question of the possible existence of two host-associated species inside the genus *Dipylidium*.

## Conflicts of interest

The authors declare that they have no conflicts of interest in relation to this article.
